# How should artificial intelligence be used in breast screening? Women’s reasoning about workflow options

**DOI:** 10.1371/journal.pone.0323528

**Published:** 2025-05-30

**Authors:** Diana Popic, M. Luke Marinovich, Nehmat Houssami, Julie Hall, Stacy M. Carter

**Affiliations:** 1 Australian Centre for Health Engagement Evidence and Values, School of Social Sciences, Faculty of Arts, Social Sciences and Humanities, University of Wollongong, Wollongong, New South Wales, Australia; 2 The Daffodil Centre, The University of Sydney, Sydney, New South Wales, Australiaa Joint Venture with Cancer Council NSW, Sydney, New South Wales, Australia; 3 School of Public Health, Faculty of Medicine and Health, University of Sydney, Sydney, New South Wales, Australia; Universitätsklinikum Magdeburg: Universitatsklinikum Magdeburg, GERMANY

## Abstract

Studies show that breast screening participants are open to artificial intelligence (AI) in breast screening, but hold concerns about AI performance, governance, equitable access, and dependence on technology. Little is known of consumers’ views on how AI should be used in breast screening practice. Our study aims to determine what matters most to women regarding AI use in the workflow of publicly funded breast screening programs, and how women choose between workflow options. We recruited forty women of screening age to learn about AI, the Australian breast screening program, and four possible workflows that include AI – one where AI works alone, and three different combinations of humans and AI. Participants then joined one of eight 90-minute dialogue groups to discuss their normative judgements on workflow options. Women proposed four conditions on AI deployment: preserving human control, evidence to assure AI performance, time to become familiar with AI, and clearly justifying the need for implementation. These informed women’s unified rejection of AI working alone, and divided preferences across the other three workflows, as they traded off workflow attributes. Current evidence on AI performance convinced some women, but not others. Most women believed humans mitigate risk the best, so workflows should continue to be designed around them. Public breast screening services are trusted and valued by women, so significant changes require careful attention to outcomes relevant to women. Our results – women’s detailed judgements on workflow design options – are new to the research literature. We conclude that women expect that AI only be deployed to do tasks it can do well, only where necessary, and only to fill gaps that radiologists cannot meet. Advancements in AI accuracy alone are unlikely to influence all women to accept AI making final decisions, if clinicians are available to perform the same task.

## Introduction

As machine learning and the digital health environment enable greater automation via medical artificial intelligence (AI), a question arises: Is this what service users want? The ethical and system challenges in delegating clinically-related tasks to AI systems [1] suggest a need for systematic engagement with the patients and publics who depend on health services. Recent systematic and scoping reviews of the relatively new literature on patient and public views [2–6] provide some insights. Knowledge of AI is variable [2,4–6]. Knowledge about healthcare AI may be less than in other contexts [2,4]. While consumers are mostly open to healthcare AI and its potential benefits [2–6], this is contingent on AI performance [2,6], use in low-risk settings [2], human oversight [5], equitable access [6], and familiarity [6]. People may accept AI if their clinicians consider it trustworthy [5,6]. When these conditions are not met, public cautiousness [5,6] or opposition [2] may result, due to concerns about performance, privacy, governance, equity, choice, alienation or dehumanisation, deskilling, and undermining patient-professional relationships [2–6]. Patients and publics call for education and consultation [2,4], remaining patient-centred [5,6], and addressing performance, governance and ethics issues before and during implementation [3–6].

One well-advanced use-case for AI in healthcare is mammographic screening for breast cancer [7]. Screen-reading AI is deployed in some breast screening contexts, including some private practices, but was not used in Australian public breast screening at the time of this study. All Australian women aged 50–74 are invited biennially to attend publicly-funded mammographic screening for breast cancer through BreastScreen (women aged 40–49 and older than 74 years are able to attend). In BreastScreen Australia, as in many organised screening programs, images are read by two radiologists; a third reader arbitrates in the case of disagreement (some programs use a consensus approach). Published studies [8–12] exploring women’s views show understandings, hopes, and concerns about AI in breast screening are similar to those expressed about AI in healthcare generally. This includes evidence that first-time screeners may hold stronger positive and negative views of AI [11], and that women are concerned about dependence on AI [8]. Women appear to have stronger expectations regarding responsibility, accountability and governance in breast screening AI than in AI in general [8,10–12]; equitable access is also important to women [8]. We have shown women are especially concerned about how AI and radiologists are combined in breast screening systems, and about AI performance [12].

Elsewhere, we published the first stage of analysis of these data [13]. This includes: 1. Women’s self-reported knowledge; 2. Women’s expressed support for the use of AI in breast screening at three timepoints; and 3. A qualitative analysis showing four conditions that women said should be met before AI was deployed: preserving human control, evidence to assure performance, time to become familiar with the technology, and clearly justifying the need for implementation, summarised in Table 1 [13].

**Table 1:** Four conditions women imposed on AI implementation in breast cancer screening – a summary [13].

Human control and decision making is preserved	Women valued human attributes, which meant the current workflow in many programs of 2–3 radiologists screen-reading independently was highly valued as an assurance of system performance. Women wanted radiologists to continue reading mammograms, retaining oversight, control and skills. This would ensure accountability for decisions, and meaningful explanations to patients. Investment in a radiology workforce should be sustained. This finding was consistent regardless of women’s overall positive or negative sentiment towards AI in breast screening.
**Relevant, high-quality evidence shows improved accuracy prior to implementation**	Regardless of overall sentiment towards AI, women highly valued AI system performance. Many were unimpressed by the (2022) evidence of performance. Radiologists, in their view, were tried and tested; AI was not. Evidence should be relevant to practice, including trials and global case studies; some women emphasised they did not want to *be* the trial.
**Women have time to get informed and familiar with AI**	AI was generally seen as unfamiliar, and sometimes futuristic, or even unrealistic. Women sometimes ascribed this to their age or generation, and sometimes worried it might be a barrier to participation. To address unfamiliarity, women suggested transparency about AI use, slow, staged implementation, and the potential for women to opt out of AI use in their care.
**Reasons for change outweigh the costs**	We told women that AI was of interest to breast screening services because of a shortage of radiologists. Responses to this premise varied. Some women were suspicious, suggesting that cost-cutting, or simply the availability of AI, were more likely reasons. Others accepted the premise, but believed screening services weren’t adequately exploring other solutions, e.g., workforce development. Some women accepted AI as a viable solution to a radiologist shortage; even they stressed that policy environments should not over-emphasise AI or grow dependent on it, and should not permit automation or radiologist job loss simply because they could. AI should not be used just because it was available: it should be used only when there were adequate reasons to support service change, and for some, only if it was necessary to keep screening services functioning. Retaining radiologists could bring future improvements in breast screening, while AI could introduce numerous downsides: it was critical that the benefits outweighed the risks, harms and losses.

In this paper, we build on this earlier work to examine a key issue for those considering the implementation of AI in breast screening programs: how AI should be included into the screening workflow. We aimed to answer the questions:

1. What matters most to women when considering the use of AI in the workflow of publicly-funded breast screening programs?
2. How do women choose between options for including AI in breast screening workflows?

## Materials and methods

### Ethics approval

Ethics approval was provided by the Human Research Ethics Committees of the University of Wollongong (2021/067).

### Approach

We used online dialogue groups, a qualitative data collection method developed by empirical bioethics researchers. Dialogue groups engage participants in discussing scenarios, asking them to make normative judgements about options, consider the potential to change their views, and provide reasons [13].

### Participant recruitment and selection

Consistent with qualitative methodologies, we aimed to recruit diverse participants whose experience and perspectives were relevant to our research questions: in this case, those eligible to be invited to public breast cancer screening. Recruitment and selection was undertaken by Taverner Research [13], via social media and random digit dialling (see S1 Appendix and S2 Appendix). Social media advertising was on Facebook, to diverse geolocated ‘community’ pages to ensure national reach. To understand the views of screening participants, we excluded those with personal/close family member/close friend experience of breast cancer (ever), or employment in breast screening, breast cancer care or cancer control (last five years). Women with a direct experience of breast cancer are frequently excluded from studies about screening (e.g., [14]) because the experience of breast cancer changes women’s experience and perception of screening [15]. We included women who had, and had not, participated in screening in the prior four years, allocating them to different groups. Women required conversational English skills and access to a computer/tablet and secure internet connection to participate.

Informed consent was recorded orally to reduce burden on participants to print, sign and scan their written consent. After participants received the Participant Information Statement, their oral consent was recorded using a digital recorder and stored on secure University of Wollongong data systems. Oral consent was approved by the Human Research Ethics Committee at the University of Wollongong. The committee did not require consent to be witnessed. Each woman spent approximately 2.5 hours over two weeks on consent, support for IT skills, engaging with information, and a 90-minute online discussion, and were compensated $AUD150.

### Information provision and data collection

**Fig 1:**
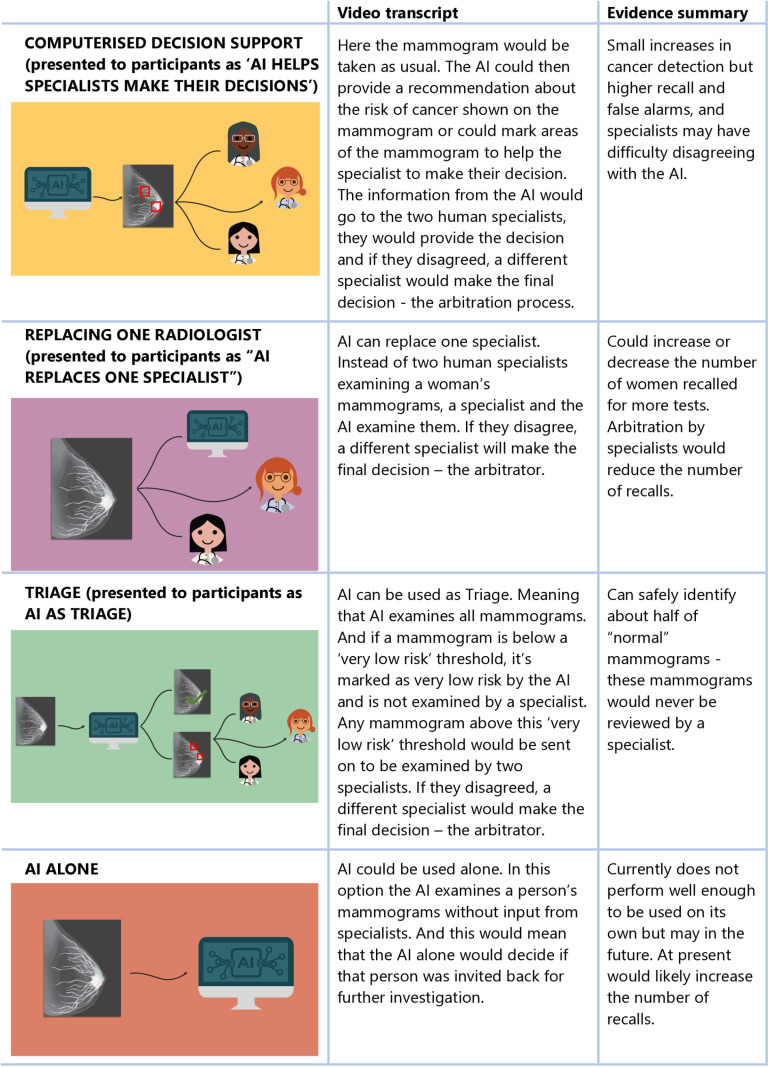
Workflow options and evidence presented in bulletin boards and dialogue groups.

Data were collected from 12 August 2022 to 21 October 2022. To support informed participation in dialogue groups, we shared three 5–10 minute videos [16–18] on specialist research-only bulletin board service VisionsLive, one every two days for six days. The videos explained: AI, including its uses; screening and breast screening, including current and potential workflows; and evidence on AI performance (as of 2022) in each workflow [19,20]. Workflows and evidence on AI performance in each is presented in Fig 1. We encouraged participant engagement and interaction. Questions women posted on the bulletin board were answered by investigators. Women were required to view all videos and post comments on at least two before participating in a group. Video content and discussion group tasks were developed between researchers and content experts in ethics, AI and breast screening.

Senior researcher SMC moderated dialogue groups by recapping information from the three videos and asking participants’ judgements on the alternative workflows, with reasons (see S3 Appendix). Audio-recordings of discussions were transcribed verbatim by a professional transcription service.

### Data analysis

Participant demographics were compared to the population of Australian women aged 50–74 using the Australian Bureau of Statistics’ Tablebuilder [21]. Dialogue group analysis focused on women’s judgements about the four workflows. We used reflexive thematic analysis (RTA) [22–26], a proven approach in qualitative health policy research offering both robustness and flexibility. DP developed codes inductively from the data, largely coded semantically and summarised patterns and insights for each group. Records of the analysis process include a spreadsheet of positive and negative judgements about the four workflows, by group and cohort, and memos to SMC for feedback. Bulletin board comments did not add to the analysis so were not included. For transparency, S4 Appendix. details our method against agreed quality standards for reporting qualitative analysis [27]. We did not measure or report theme frequency, as the authors of the method have specified that this is inconsistent with the tenets of RTA [24].

## Results

### Participant characteristics

We recruited eight groups of 4–6 women, totalling 40 participants. We achieved diversity across age groups, residential location, levels of education (from early school leaver to postgraduate), most states and territories, and birthplace in or outside of Australia. Groups contained proportionally more younger and university-educated women, and more women born in Australia, compared to the Australian population. More demographic details are available in our earlier paper [13].

### Openness to AI comes with conditions

Women commonly expressed the following: ‘*yes* [I’m open to the development of AI in breast screening], *but* [on conditions]’. As we will show, women’s judgments about the four workflows relied heavily on the four conditions reported in our introduction [13]. Women were open to potential benefits, but also recognised that contemporary life featured extensive, disappointing automation. Women not screened in the last four years appeared overall to be more cautious about AI than women who had screened. However, their substantive concerns were similar, so they are not reported separately in our analysis.

### Women’s judgements about four potential workflows for AI in breast screening

The four conditions in Table 1 informed women’s judgements about the four potential workflows they considered. Below we show how women explained their negative and positive judgements about these workflows.

### Different women preferred different workflows

**Fig 2:**
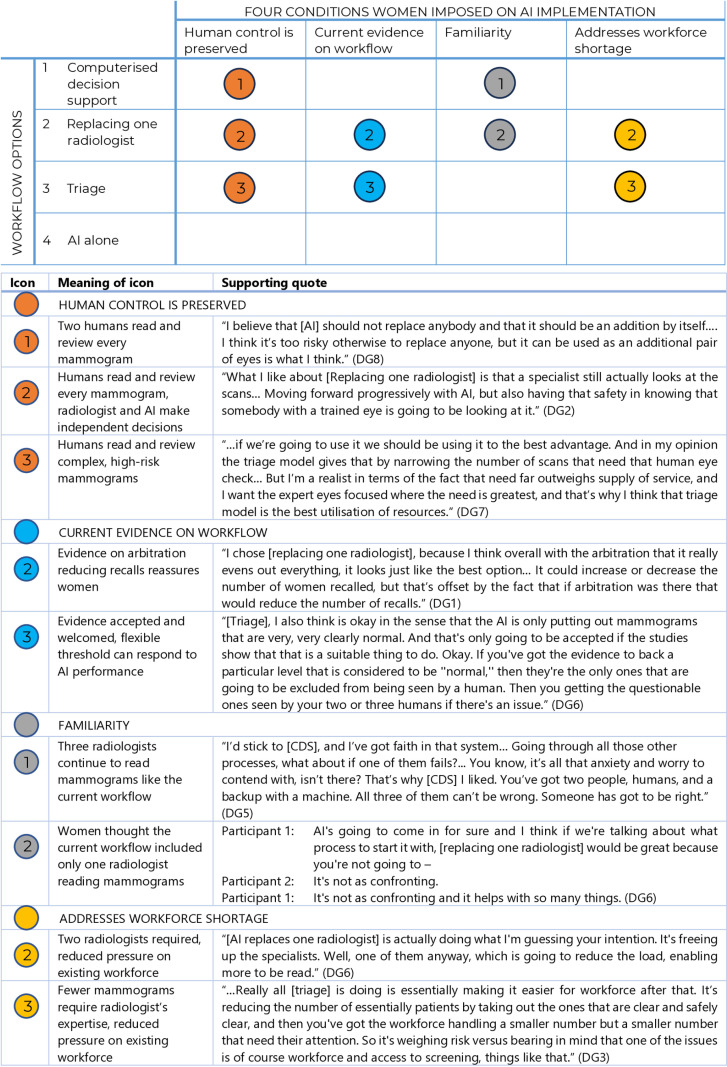
Women’s positive judgements about potential workflows for AI in breast screening.

**Fig 3:**
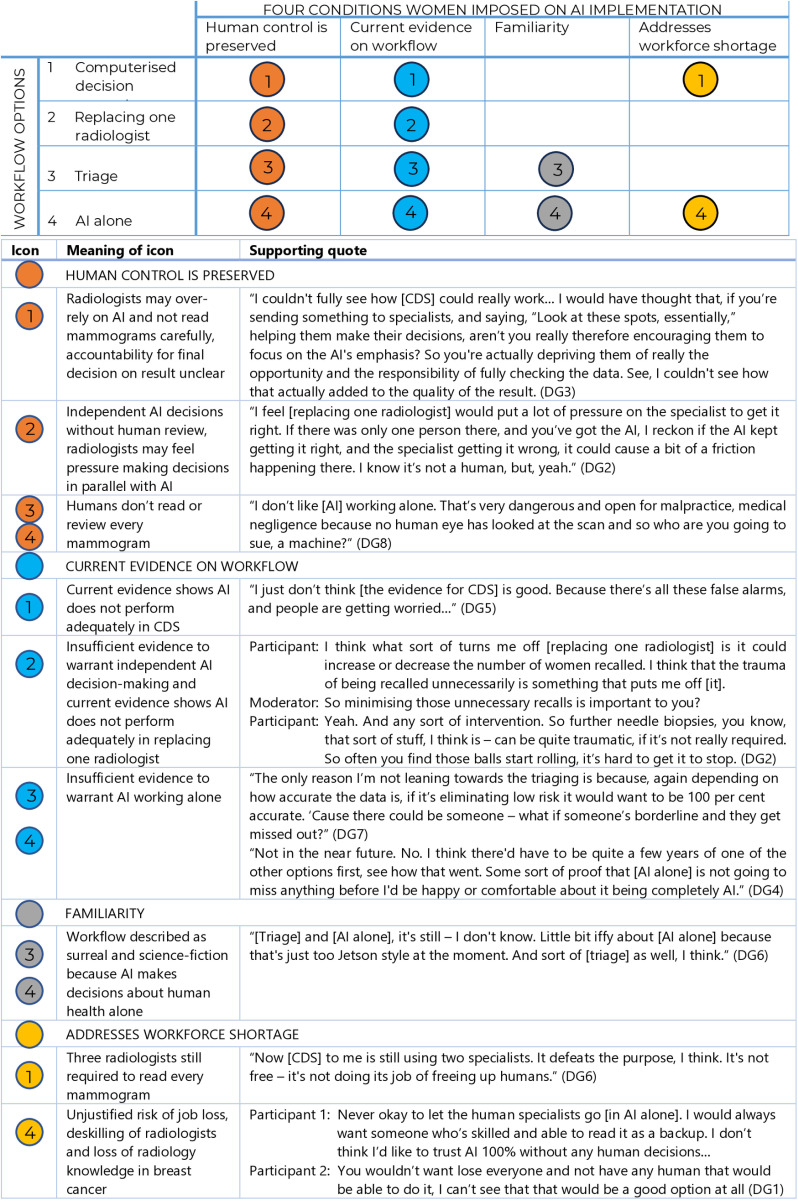
Women’s negative judgements about potential workflows for AI in breast screening.

Women consistently rejected using AI alone. However, judgements about the other three workflows – Computerised Decision Support (CDS), replacing one radiologist, and triage—were divided. Women sometimes changed their views, had difficulty deciding, or disagreed. Figs 2 and 3 illustrate women’s most common responses in relation to their four conditions.

### Computerised decision support (CDS)

Women who preferred CDS emphasised the importance of human control and familiarity. They supported CDS because two radiologists continued to read every mammogram, with AI relegated to being a complementary tool used by humans. Familiarity—resemblance to the current workflow—was also reassuring. These women were mostly unconvinced by the evidence presented. When pushed on what they thought about the potential for CDS to increase false positives, some said the risk would be mitigated by the two or three humans.

The women who rejected CDS, conversely, were concerned it would undermine human decision-making: *independent* human decision-making was valued, to preserve human control and accountability. They were more concerned about evidence CDS could increase false positives, and worried that CDS would not address the workforce shortage.

### Replacing one radiologist

Women who preferred this workflow also reasoned from familiarity—because this workflow appeared similar to the current one—and human control, because every mammogram was seen by at least one human. Women saw this workflow as a reasonable trade-off: every mammogram was seen by a radiologist, but with fewer radiologists than the current workflow, thus addressing workforce shortages. With respect to evidence, some of these women were unmoved, others were reassured by evidence that arbitration could reduce false positives. This workflow was seen to combine the best attributes of AI and humans.

Some women—mostly those preferring CDS—said there was insufficient evidence to warrant AI making any decisions independent of humans. Using AI to read independently would require evidence that it was 100% accurate.

### Triage

Triage generated the widest range of views. This turned in part on what it means to retain human control and human decision making. Women who favoured triage said this need not mean all mammograms being read by a person: it could instead entail directing human expertise and resources to complex, high-risk mammograms. These women valued the capacity for programs to set a flexible, performance-based threshold, ensuring only very low-risk mammograms would be read by AI alone, while human expertise could be applied to higher risk mammograms, where it was most needed.

Women who preferred triage also appeared more likely to reason from the evidence, and while they accepted and gave weight to evidence on all four workflows, they favoured the evidence for triage. They also preferred triage as a solution for workforce shortages.

In contrast, some women disliked or strongly opposed an AI system triaging mammograms out of radiology review. To these women, maintaining human control meant preserving human decision making over *every* mammogram. They saw the evidence as insufficient to warrant AI making any final decisions and tended to reject or question the current evidence. For quality assurance or to improve acceptability, some women suggested human review of a subset of AI-read low-risk mammograms. Triage was also rejected as too unfamiliar—even “sci-fi”—by some women.

### AI alone

Women rejected AI working alone. The evidence suggested it did not yet perform well enough for this use; it was also ‘too out there’, futuristic and unfamiliar. Some said AI alone may be acceptable in the distant future (10 or 20 years away); others said it would never be acceptable or justified. This was because of loss of human control, radiologist job loss, loss of radiology skills and knowledge, and loss of human accountability.

### Patterns of judgments across the workflows

Across the four workflows, patterns of intuition and judgement help explain women’s choices.

### Women trade workflow attributes against each other

Women chose workflows by weighing up how well each met their four conditions. Each woman weighted each condition differently. When multiple conditions were equally important, choosing a preferred workflow became more difficult.

Some women perceived AI implementation as inevitable, thus chose workflows that they thought health services would consider ‘realistic’, even if they were unfamiliar, discomforting, or non-preferred. This is a reminder that women may express acceptance of a workflow but not be enthusiastic for the workflow, instead reluctantly conceding industrial, cultural, and technological change.

*Moderator: What’s your thought about [AI replaces one specialist]?*

*Participant: Look, I go back to that’s fine, but I still think it’s got maybe a bit to do with the shortage of medical staff, qualified medical people. I guess inevitably AI is going to come in whether we like it or not, but to what degree. (DG1)*

### Most women accept a workforce shortage as reason to introduce AI

Many women took seriously the shortage of radiologists; those more open to this premise typically preferred either replacing one radiologist or triage. Conversely, women preferring CDS tended not to discuss workforce shortages, so may have seen this as insufficient justification to override other conditions.

### AI making final decisions was a change too far for most women

Familiarity interacted with human control/decision making. The least familiar workflows were triage, and AI alone. These were described as science-fiction—allowing AI to make final decisions—thus required the greatest leap in reimagining how health status was decided. Women prioritising familiarity preferred CDS or replacing one radiologist, which represented the least change in who interpreted their mammograms.

### Current evidence influences some women’s preferences, but not others

Women were invited to consider whether their preferences had changed based on the evidence on AI’s performance. Some women were unmoved, staying with their preferred workflows, typically CDS or replacing one radiologist. Here, human control and familiarity were often more important than AI performance, diminishing the impact of evidence.

Women seemed most likely to use evidence when they were already open to a workflow for other reasons. If women believed humans should read every mammogram, evidence of safety did not sway them towards triage. In contrast, if women were open to most workflows, and to the evidence, they often preferred triage, followed by replacing one radiologist.

Women debated whether false positives or false negatives were worse: fear about both scenarios influenced their decision-making. The more women wanted to avoid false positives, the more likely they were to prefer triage or replacing one specialist, based on the evidence provided.

Women were asked how accurate AI needed to be for women to have faith in it: some accepted AI performing as well as radiologists, others said it needed to perform better; some nominated 95–99% accuracy, others 100%. Women responded with the same *range* of views, whichever workflow they considered.

### Humans mitigate risk the best, so workflows should be designed around them

All women had greater confidence in radiologists than they did in AI to read mammograms and mitigate risk. The shared view that evidence for AI was insufficient likely reinforced confidence in humans. Although researchers presented the workflows in relation to the role of AI, women centred human attributes, and gave most weight to the role of *humans* in each workflow. However, as shown, different women approached the relationship between risk and human mitigation differently. For some, every mammogram was at equally high risk of an inaccurate result: thus, humans should read every mammogram to mitigate this risk, and CDS or replacing one radiologist were preferred. Others saw only a subset of complex mammograms as high risk: thus, AI as triage was preferred to focus radiologists’ attention on these high-risk mammograms.

## Discussion

There is a small and growing literature on women’s views on the use of AI in breast screening, which we have reviewed elsewhere [13]. To our knowledge, this is the first study of the views of women of screening age about workflow design options for AI in breast screening. Women’s judgements turned on four conditions: retaining human control and decision making, high-quality evidence of excellent AI performance, familiarity, and clear reasons for change. The first two conditions are reflected in existing systematic reviews of consumer views of AI in healthcare [2–6]. The second two conditions are less prevalent in systematic reviews, so may be particularly relevant to breast screening. They may be explained in part by the cohort (older women are reportedly sometimes more apprehensive about healthcare AI [28]) and our methodology (we began from a premise for introducing AI in screen-reading). Women rejected use of AI alone in mammography screen-reading and made different judgements about three other workflows (CDS, replacing one radiologist and triage) depending on how they weighted the four conditions. These detailed judgements about the four workflows are new to the literature.

Inevitably, AI performance will improve. Women for whom accuracy is most important, if their accuracy expectations are met, are likely to welcome these advancements and be most open to expanding AI’s role. But it would be wrong to assume that most women would welcome AI unconditionally. For women who value radiologists, accountability, screening services contributing to employment and the advancement of human skills and knowledge, it will be harder to accept a larger role for AI. Different women prioritise different conditions, and so make different judgements. However, it seems likely that improved accuracy will not lead all women to accept AI making final decisions, if clinicians are available to perform the same task.

### Strengths and limitations

Limitations of our study include that the participants were on average somewhat younger, and more university educated and more likely to be Australian-born, than the broader population of Australian women. Despite this, we achieved age diversity, majority non-university educated participants, and 28% overseas born, so our findings reflect diverse women’s views. Another limitation is existing uncertainty about AI implementation in practice (e.g., regarding accountability, accessibility, speed of results), such that the conversations were necessarily somewhat speculative. As AI technology is evolving quickly, recent evidence about AI performance [7] is not captured in the information presented to participants. The fact that women had an opportunity to learn about breast screening and AI before the discussion groups is a strength: we note that they contain the kind of information that might be required to support informed consent to participate in breast screening using AI, so the results of this study are arguably more relevant to practice than collection of uninformed views. We have made the videos public to allow readers to make their own assessment of the information presented and its likely effect on women’s views; we also hope the videos may be useful in other contexts. Although there may be some selection bias in the sample (e.g., due to recruitment of some women via social media, and the online format), we note that in Australia more than 90% of the population use social media and internet respectively [29]. As in all group-based qualitative research, group dynamics may have had some impact on the findings, although we note that the facilitator is an experienced qualitative researcher who aimed to mitigate such impacts as much as possible (for example, ensuring that all participants were able to speak).

There is some evidence that when patients consider AI in their own healthcare, they are more hesitant to its utilisation [28]. A novel strength of this study is our presentation of concrete and likely implementation scenarios, allowing women to consider these against their own experience, values and expectations.

### Implications for policy and research

Breast screening is a complex public health service producing a range of benefits and harms for women, requiring complex decision making about service objectives and design. The balance of outcomes (lives lengthened by early intervention, false negatives and positives, cancer overdiagnosis) is a matter of ongoing debate.[30] Future screening modalities are also in flux, including possible future introduction of polygenic risk scoring [31]. The potential introduction of AI is one more element in this complex policy landscape [32]. However, for the women who participate, breast screening is both a trusted and a valued service [33]. This means significant changes—such as the introduction of AI screen reading—require careful attention to outcomes relevant to women and to screening policymakers [34]. There was a clear message in this study that women expected to be treated as active stakeholders in these decisions. As public knowledge about the potential risks and harms of AI grows, we suggest that services should think carefully about what is required both for public engagement and individual patient consent in the use of AI.

## Conclusion

We have shown that women’s judgements about workflows for AI in breast screening rely on different interpretations and combinations of four conditions: preserving human control and decision making; availability of relevant, high-quality evidence of performance; familiarity; and being convinced that the reasons for change outweigh the potential harms, risks, or costs. Although different women preferred different workflows, all rejected AI working alone in reading mammograms. Looking across the conditions, we conclude that women would ask that AI only be deployed to do tasks it can do well, only where necessary, and only to fill gaps that radiologists cannot meet.

## Supporting information

S1 Appendix Demographic and screening questions. (DOCX)

S2 Appendix Social Media Advertisement. (DOCX)

S3 Appendix Moderator guide. (DOCX)

S4 Appendix Standards for reporting qualitative research checklist. (DOCX)
