# Supplementary material for: How should artificial intelligence be used in breast screening? Women’s reasoning about workflow options
Source: PLoS One. 2025 May 30;20(5):e0323528. doi: 10.1371/journal.pone.0323528 (PMC12124851; doi:10.1371/journal.pone.0323528)
Supplement: S1 Appendix — (DOCX) [file pone.0323528.s001.docx]

# S1 Appendix: Script for Random Digit Dialling and preliminary recruitment of dialogue group participants

**INTRODUCTION**

*Hello this is [Name] from [Taverner Research] calling on behalf of researchers at the University of Wollongong. I am ringing to see whether you or someone in your household might be interested in participating in a research project about the use of artificial intelligence in breast screening. Researchers are looking for women aged 50-74 for the study.*

*Are there any women in your household who are in that group?*

IF **NO**: Thank and end call.

IF **YES but you are not speaking with that person**:

Ask to speak to eligible person OR Record name of eligible person and best time to call back: ­­­­­­­­­­­­­­­­­­­­­­­­­­

IF **YES and you are speaking with eligible person:**

*[If needed: Hello this is [Name] from [Taverner Research] calling on behalf of researchers at the University of Wollongong.]*

*I am ringing to see whether you might be interested in participating in a research project about the future use of artificial intelligence in breast cancer screening. Researchers are seeking women aged 50 to 74 for the study. Could I take 5 minutes to tell you a little more about the project?*

**IF NOT INTERESTED:** Thank and end call

**IF INTERESTED BUT CAN’T TALK NOW:** Thank and arrange time to call back:

**IF INTERESTED AND ABLE TO TALK NOW:**

*Researchers from the Australian Centre for Health Engagement, Evidence and Values at the University of Wollongong are seeking participants for a study about future uses of artificial intelligence in breast cancer screening.*

*If you were to participate, there are three components of the research.*

*The first will be a bulletin board. Over two weeks, you would look at 3 short videos about AI and Breast screening. You will be asked to post comments about at least two of the videos as part of your participation on the bulletin board.*

*The second component will be an online discussion group of about 1½ hours with 4-5 other women.*

*You would also be asked to do a 3 minute survey 3 times - at the very beginning, just before the discussion group, and at the very end.*

*The discussion groups and the Bulletin Boards will be held on a research platform called VisionsLive which will be easily accessible to you. There will be a number of groups, to help find a time that’s convenient for you.*

*On the bulletin board, you will hear about possible ways that artificial intelligence could be used in breast screening in future.*

*In the discussion group, you will be asked to talk about whether you think that these alternatives would be a good or a bad thing.*

*In our experience, people who participate in a dialogue group find it interesting and rewarding.*

*All participants will receive a $150 gift voucher at the end of the project. To get the voucher you would need to provide a comment on at least two of the three short videos, and attend one discussion group, over the course of two weeks. We would expect this would take you about two and a half hours in total, spread over two weeks.*

*Is this something that you might be interested in being involved in?*

IF NO - Thank and end call

IF YES OR MAYBE

Caller:

*OK great… So in order to assess your eligibility I need to ask you a few questions. Is that OK?*

1. Have you ever worked in breast screening or breast cancer care OR a cancer control agency (for example Cancer Council or NSW Cancer Institute) in the last 5 years? IF YES, RESPONDENT IS NOT ELIGIBLE: *Thank you, this study is not including people who have worked in breast screening or breast cancer care recently. Exit call.*

*I have two additional eligibility questions for you, which are a little personal I’m sorry –*

1. Have you ever been diagnosed with breast cancer or DCIS? (Yes/No).

IF YES, RESPONDENT IS NOT ELIGIBLE: *Thank you, this study is not including people who have experienced a breast cancer diagnosis. Exit call.*

1. Has a close relative or a close friend- of yours ever been diagnosed with breast cancer or DCIS (Yes/No). By close relative we mean mother, sister, daughter, aunt, or grandmother. IF YES, RESPONDENT IS NOT ELIGIBLE: *Thank you, this study is not including people with a close relative who has experienced a breast cancer diagnosis. Exit call.*

*Sorry to have to ask such personal questions – we asked because we want to be sure we are being careful about women’s experience going into the project*

**CALLER PLEASE NOTE:**

*If any women seem concerned about the question, please ask the women whether they would like information about support services and that we recognise that there may be topics raised in the dialogue groups that participants may find upsetting.*

For specific information and advice about breast screening, you can contact Breast Screen Australia on: PH: 1800 020 103,

https://www.health.gov.au/initiatives-and-programs/breastscreen-australia-program

For specific information and advice about cancer, or for emotional or practical support for people living with cancer and their friends and family, you can contact the Cancer Council in your state on 131120. More information is available at <https://www.cancer.org.au/support-and-services/cancer-council-13-11-20>

*Thank you. Now I just have a few extra questions about you*

1. *Have you ever had breast cancer screening? (Yes/No/Unsure)*

*IF UNSURE: Breast screening is where the radiographer puts a plate on the top and the bottom of your breast and takes an X-ray picture. Can you remember whether you have had that done at some stage?*

*IF YES: Can you remember roughly how many years ago you had your latest screen (approximate number of yrs ago)*

*Can you remember whether it was with the free government BreastScreen program or with a private breast screening service?*

1. *…………………………………………………[note whether BreastScreen or Private]*

*OK just a few more questions*

1. How old are you? ___
2. What is your postcode? ____
3. How long have you lived in that area?
4. What is your country of birth?
5. Do you identify with a culture other than the culture of your country of birth?
6. Are you working at the moment?
   1. Working fulltime c. Retired
   2. Working part-time / semiretired d. Unemployed
7. What did you / do you do for work? __________________
8. What is your highest qualification?
   1. School Certificate d. Undergraduate degree
   2. High School e. Postgraduate degree
   3. Trade Certificate

*Caller:*

1. Thank you. Are you happy for me to give your contact information to the research team at the University of Wollongong so they can discuss the project with you / send you some more information?

**IF NO:** Thank you very much for your time. Good bye.

**IF YES:** Thank you. Not everyone will be eligible and not everyone who is eligible will be able to be invited. The researchers will be in contact with you as soon as possible about your possible participation. What is the best way to contact you?

Phone: _____________________

Email: ___________________________

If you are invited to participate, we will let you know, and you will receive a **call from the researchers** about 2 weeks before the group. They will explain everything that is going to happen in more detail, and help you get started on the research platform VisionsLive.

**Thank you and goodbye**
