# Supplementary material for: How should artificial intelligence be used in breast screening? Women’s reasoning about workflow options
Source: PLoS One. 2025 May 30;20(5):e0323528. doi: 10.1371/journal.pone.0323528 (PMC12124851; doi:10.1371/journal.pone.0323528)
Supplement: S2 Appendix — (DOCX) [file pone.0323528.s002.docx]

**S2 Appendix: Social Media Advertising Text to be used by Taverner Recruitment in a targeted Facebook campaign**


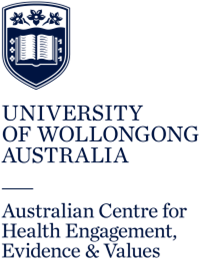


**Women’s perspectives on the use of Artificial Intelligence (AI) in Breast Cancer screening**

Participate in online research: 2 hour online group discussion in (month). Gift voucher $80 as thanks for your participation.

For details and to apply go to the link: [insert link to online screener]
Strictly research only - your details are NOT shared with anyone outside this project.
