# Supplementary material for: How should artificial intelligence be used in breast screening? Women’s reasoning about workflow options
Source: PLoS One. 2025 May 30;20(5):e0323528. doi: 10.1371/journal.pone.0323528 (PMC12124851; doi:10.1371/journal.pone.0323528)
Supplement: S3 Appendix — (DOCX) [file pone.0323528.s003.docx]

# S3 Appendix: Moderator guide for dialogue groups on AI use in potential workflows for breast cancer screening

Table S1: Moderator guide for dialogue groups on AI use in potential workflows for breast cancer screening

| **Time: 0** | **Introduction (5 mins)** |
| --- | --- |
| **SECTION 1** | Background, conditions for consent, how meeting will proceed |
| **Time: 5 mins** | **Introducing you (10 mins)** |
| **SECTION 2** | Icebreaker activity |
|  | **Ground rules** |
|  | - The focus now is on discussion - No right or wrong answers, expect different perspectives, will ask why to understand your reasons, try not to talk over one another, show respect for one another |
| **Time: 15 mins** | **Today we are talking about artificial intelligence in breast screening (5 mins)** |
| **SECTION 3**  **Slides from online presentation** | Quick recap of some of the key information from the three videos that you viewed - show brief presentation (2-3 very brief slides)   - How does breast screening work right now?   - Women aged 50-74 invited every two years   - Every mammogram examined by two specialists, and if they disagree, by a third specialist.   - AI is not currently being used to read mammograms in Australia - Why might we want to use AI to read mammograms?   - Reasons on slide (shortage of specialists, work capacity of AI, hope that AI might do better than specialists one day – but as you heard, this is complicated!) - This discussion will focus on *how* might we use AI in breast screening, and *what do you expect* from AI if it’s to be used in breast screening? |
| **Time: 20 mins** | **First impressions (15 mins)** |
| **SECTION 4** | Let’s begin with a general discussion: now that you’ve watched all the videos and interacted, what are you thinking about using AI in breast screening? |
| **Time: 35 mins** | ***HOW* SHOULD WE USE AI IN BREAST SCREENING? (20 minutes – 5 for ranking and 15 for discussion)**  **You learned about different ways AI could be used**  **We want to compare these**  **Which do you think are better, which are worse, and why** |
| **SECTION 5**  **Slide with options from online presentation** | Re-introduce the options with the graphic from the video: AI triage, AI replaces one specialist, AI helps specialists make their decision, only AI |
|  | **Prompt: We want to know what you think are the best and worst things about these options.**  Please use this chat function to list good and bad things about the four options. List the option number first (1, 2, 3 or 4), then write something you like or don’t like about that option, then press the ‘post’ button (the paper airplane symbol). You can post as many times as you like. |
|  | Give them 5 minutes to add positive, negative and neutral comments in the chat |
|  | **Open up for discussion**:  The good things – what did you like about these options?  The worse things – what didn’t you like about these options?  **Throughout, keep asking about reasons – why do you think it’s good or not good?** |
| **Time: 50 mins** | **HOW ACCURATE DOES AI IN BREAST SCREEN NEED TO BE? (25 mins – 5 + 5 + 15)** |
| **SECTION 6**  **Slides from online presentation** | Now we are going to discuss what we know about the accuracy of AI in each of these 4 workflow options  Recap evidence summaries  Show two slides:   - Things that could happen to Nadia - Summary of accuracy of four options |
| **SECTION 8**  **Discuss** | **15 MINS**  **Ask them to vote by raising their hand – most and least preferred**  **Discuss– draw out reasons**   1. **Most favoured** 2. **Least favoured** 3. **Others.** |
| **SECTION 9** | **5 MINS - AI ALONE**  **Go back to outcomes for Nadia slide**  **Remember that you don’t meet the specialist – they sit in a room and read the mammograms**  **How good would AI have to be to accept using AI alone?** |
| **Time: 80 mins** | **CLOSING DISCUSSION : AI BREASTSCREEN SCENARIO (10 MINS)** |
|  | Think about everything we’ve discussed. Imagine Rachel, Breast Screen Australia Program manager, has to make a decision about whether to introduce AI into BreastScreen services, and if so, how. |
|  | What message would you give to Rachel as she tries to decide about whether (or how) to introduce AI into BreastScreen? |
| **Time: 90 mins** | **FINAL SURVEYS**  **Thank you and any final comments – we’ll send your vouchers very soon by email** |
