# Supplementary material for: How should artificial intelligence be used in breast screening? Women’s reasoning about workflow options
Source: PLoS One. 2025 May 30;20(5):e0323528. doi: 10.1371/journal.pone.0323528 (PMC12124851; doi:10.1371/journal.pone.0323528)
Supplement: S4 Appendix — (DOCX) [file pone.0323528.s004.docx]

# S4 Appendix: Detailed qualitative method as per standards for Reporting Qualitative Research^1^

Table S2: Detailed qualitative method as per standards for Reporting Qualitative Research^1^

| Qualitative approach and research paradigm | Bulletin Board activities allowed participants to view and engage with video information about AI and breast cancer screening before their online dialogue group. See main manuscript for more details. |
| --- | --- |
| Researcher characteristics and reflexivity | SMC is a senior social scientist, public health researcher and methodologist, with 25 years of qualitative research experience. She has worked on applied ethics projects about population screening, including breast cancer screening, for more than a decade, and on ethical and social implications of healthcare AI for five years. She led the project and facilitated the groups.  DP is a qualified anthropologist and experienced qualitative researcher in various social fields. She has no previous experience researching breast cancer or AI, has no life experience of breast cancer, and is not of screening age. She holds no assumptions about the research topic that could influence the study’s results and knows none of the participants. She documented her reflexive thematic analysis, including reasons for methodological decisions, and regularly shared these with SMC for comment.  JH worked on this project as a senior research assistant, and has a background in social research and public health. She was instrumental in managing the project and organising the groups, and acted as an assistant moderator to provide support to participants as needed throughout the group activities.  LC worked on this project as a research assistant and has a background in public health. She was the point of contact for participants during the consent process. She acted as an assisted moderator to provide technical support to participants if needed.  MLM is a public health researcher and epidemiologist. He has more than 20 years’ experience in the evaluation of new health technologies, with a focus on tests for breast cancer screening, staging and surgical planning. For the last five years, he has led a research program evaluating the accuracy of AI for interpreting mammograms in real-world population screening settings. He contributed to developing study concepts and acquired funding for the research.  NH is a clinician and a Public Health physician – she has 30 years’ experience in breast screening and diagnostic services and leads a breast cancer research program at the University of Sydney. She has expertise in evaluation of population screening for breast cancer, focusing on assessing the benefits and harms and the clinical impact of using tests and new technologies. She contributed to developing study concepts including clinical content. |
| Context | See main manuscript. |
| Sampling strategy | Women required access to a computer/tablet to participate because VisionsLive was not accessible on mobile phones. They also required conversational English skills, as we could not offer simultaneous translation. This project required a relatively homogeneous population (all women in a limited age range). We drew on the literature^2^ and decided that eight dialogue groups would provide sufficient data for analysis and to draw conclusions. See main manuscript for more details. |
| Ethical issues pertaining to human subjects | Verbal consent was recorded for each participant. See main manuscript more details. |
| Data collection methods | The research team monitored participation in the bulletin boards and provided two email prompts to participants who did not engage with the materials. Participants who did not engage were replaced by Taverner. Dialogue groups were supported assistant researchers JH and LC. See main manuscript for more details. |
| Data collection instruments and technologies | VisionsLive chat function was used to record women’s workflow preferences because participants found it difficult to use the heatmap tool on VisionsLive. Audio-recordings of discussions were recorded by VisionsLive. |
| Units of study | See main manuscript. |
| Data processing | All research data is securely stored on University of Wollongong data systems and only research staff working on the project with authorised approval can access the data. |
| Data analysis | We used reflexive thematic analysis (RTA),^3-7^ enabled us to explore the dataset from new vantage points during analysis and incorporate concepts of consumer choice.  We developed codes inductively from the data and added codes from our previous, related study,^8^ and from AI ethics, AI policy and breast screening literatures. We largely coded semantically (mapping what women said directly, rather than looking for latent ideas). Transcripts were coded twice, in a different order each time (to prevent focus on what was most recently read), and once whilst listening to audio recordings (to understand meaning in the way participants raised opinions). Codes were refined after the first round.  After coding each transcript, DP summarised insights from that group. After coding was completed, DP wrote a summary memo to identify patterns and compare cohorts (screened/not screened last four years); DP also mapped positive and negative judgements about the four workflows, by group and cohort, in a spreadsheet, to examine the popularity of the four workflow options. SMC provided feedback on coding structure, process decisions, memos, and analysis at each step.    DP produced the final themes and subthemes by clustering codes and mind mapping, with input from SMC. See main manuscript for more details. |
| Techniques to enhance trustworthiness | DP kept running records of the analysis process and her reflexive practice for an audit trail. DP triangulated and tested themes and subthemes by writing draft abstracts and reordering transcripts according to subtheme headings. This resulted in some reorganisation of themes to better reflect what participants focused on. |

## References

1. O'Brien BC, Harris IB, Beckman TJ, Reed DA, Cook DA. Standards for reporting qualitative research: a synthesis of recommendations. Acad Med 2014;89(9):1245-51. (In eng). DOI: 10.1097/acm.0000000000000388.

2. Guest G, Namey E, Mckenna K. How Many Focus Groups Are Enough? Building an Evidence Base for Nonprobability Sample Sizes. Field Methods 2016;29(1):3-22. DOI: <https://doi.org/10.1177/1525822X16639015>.

3. Braun V, Clarke V. Thematic Analysis: A Practical Guide. London: SAGE Publications, 2022.

4. Braun V, Clarke V. One size fits all? What counts as quality practice in (reflexive) thematic analysis? Qualitative Research in Psychology 2021;18(3):328-352. DOI: 10.1080/14780887.2020.1769238.

5. Braun V, Clarke V. Reflecting on reflexive thematic analysis. Qualitative Research in Sport, Exercise and Health 2019;11(4):589-597. DOI: 10.1080/2159676X.2019.1628806.

6. Braun V, Clarke V. What can "thematic analysis" offer health and wellbeing researchers? International Journal of Qualitative Studies on Health and Well-being 2014;9:26152. DOI: 10.3402/qhw.v9.26152.

7. Byrne D. A worked example of Braun and Clarke’s approach to reflexive thematic analysis. Quality & Quantity 2022;56(3):1391-1412. DOI: 10.1007/s11135-021-01182-y.

8. Carter SM, Carolan L, Aquino Y, et al. Australian women's judgements about using artificial intelligence to read mammograms in breast cancer screening. DIGITAL HEALTH 2023;9. DOI: 10.1177/20552076231191057.
